# Dual-Speed Reassembly of Soil Microbial Networks Under Intensive Ornamental Planting: Divergent Stability Strategies of Bacteria and Fungi in Botanical Garden Cinnamon Soils

**DOI:** 10.3390/microorganisms14040865

**Published:** 2026-04-11

**Authors:** Tai Gao, Dakang Zhou, Baibing Wang, Ruifeng Wang, Gan Xiao, Han Quan, Yu Wei

**Affiliations:** 1 Beijing Botanical Garden Management Office, Bejing 100093, China; gaotai36@outlook.com (T.G.); zdk0812@163.com (D.Z.); wbb_bill@126.com (B.W.); feng952525@163.com (R.W.); xiaogan@chnbg.cn (G.X.); wangyindz@sina.com (H.Q.); 2 Beijing Floriculture Engineering Technology Research Center, Bejing 100093, China; 3 Key Laboratory of National Forestry and Grassland Administration on Plant Ex Situ Conservation, Bejing 100093, China

**Keywords:** ecological resilience, management disturbance, rotation, co-occurrence analysis

## Abstract

Intensive ornamental planting is increasingly prevalent in urban green spaces, yet its effects on soil microbial community assembly and interaction networks remain poorly understood. Here, we examined shifts in soil properties, microbial diversity, community composition, and interaction networks across successive planting cycles. Bacterial alpha-diversity remained relatively stable, whereas fungal communities showed pronounced sensitivity to early planting stages. Beta-diversity analyses revealed that bacterial community composition was jointly influenced by planting stage and site type, while fungal communities were primarily structured by site characteristics. Co-occurrence network analysis revealed contrasting reassembly trajectories between microbial groups. Bacterial networks exhibited increasing complexity and modularity, indicating enhanced interaction intensity and competitive structuring under intensive management. In contrast, fungal networks displayed reduced connectivity but maintained or recovered modular organization, suggesting structural buffering. Notably, keystone taxa remained taxonomically conserved, indicating that network reorganization was driven by interaction rewiring rather than species turnover. We propose a dual-speed reassembly framework in which bacteria function as fast-responding components with dynamic interaction networks, whereas fungi act as slow-buffering, structurally persistent elements. This decoupling of short-term functional responsiveness and long-term stability provides new insights into how intensive management reshapes soil microbiomes in botanical garden ecosystems.

## 1. Introduction

Soil functions as a central integrator within plant–soil–microbe systems, regulating nutrient cycling, biodiversity maintenance, and ecosystem stability through tightly coupled physicochemical and biological processes [1]. Among soil biota, microorganisms are primary drivers of ecosystem functioning, mediating organic matter decomposition, nutrient mineralization, and plant–microbe feedbacks that influence plant productivity and health [2,3].

Agricultural and horticultural management practices profoundly reshape soil environments by altering nutrient availability, disturbance regimes, and plant community composition [4]. While monocropping and intensive multi-cropping systems enhance land-use efficiency, they may also induce continuous-cropping obstacles, microbial imbalance, and nutrient-driven shifts in community assembly [5,6]. Most previous research has focused on agricultural soils, where management intensity is embedded within relatively stable land-use contexts [7].

In contrast, intensively managed ornamental soils in botanical gardens represent a distinct ecological scenario. Ornamental systems in botanical gardens are characterized by high-frequency plant replacement, short-term crop occupation, repeated tillage, and substantial external inputs [8]. These features generate recurrent resource pulses and disturbance events that differ fundamentally from traditional agricultural rotation systems [9]. Yet, despite the rapid expansion of urban green spaces worldwide [10], the mechanisms by which long-term horticultural management in botanical gardens reshapes microbial assembly processes and interaction networks remain poorly understood [11].

Community assembly theory predicts that environmental filtering and disturbance regimes can differentially structure microbial guilds with contrasting life-history strategies [12]. Bacteria, with high metabolic plasticity and rapid turnover rates, are expected to respond sensitively to short-term resource fluctuations [13,14]. Fungi, by contrast, possess spatially integrated hyphal networks that may buffer against localized disturbance and encode longer-term habitat filtering [15]. However, empirical evidence directly comparing bacterial and fungal responses under long-term ornamental management is scarce [11].

The former Beijing Botanical Garden, now known as the China National Botanical Garden (Northern Garden), contains a long-term intensively managed ornamental planting area. Subjected to over 22 years of recurrent tulip–summer annual–chrysanthemum succession, this area provides an ideal system to examine these questions. This site offers a rare opportunity to disentangle how long-term horticultural disturbance influences soil physicochemical properties, enzymatic activities, microbial composition, and interaction networks within the same environmental context.

In this study, we characterized bacterial and fungal communities across successive ornamental planting stages using high-throughput amplicon sequencing and compared them with relatively low-disturbance lawn-tree soils. By integrating soil physicochemical indicators, enzyme activities, community composition, and co-occurrence network analysis, we aimed to:

(1) Determine how long-term intensive ornamental planting alters microbial community reassembly (i.e., disturbance-driven reorganization of community structure and interaction networks) across planting stages;
(2) Evaluate whether bacterial and fungal communities exhibit divergent reassembly trajectories under long-term disturbance;
(3) Identify mechanistic linkages between soil properties, enzymatic activity, microbial interaction networks, and soil health in botanical garden ecosystems.

By addressing these questions, this study advances understanding of how high-intensity horticultural management reorganizes belowground ecological processes and contributes to the resilience or vulnerability of botanical garden soil systems.

## 2. Materials and Methods

### 2.1. Study Site and Management Regime

**Figure 1:**
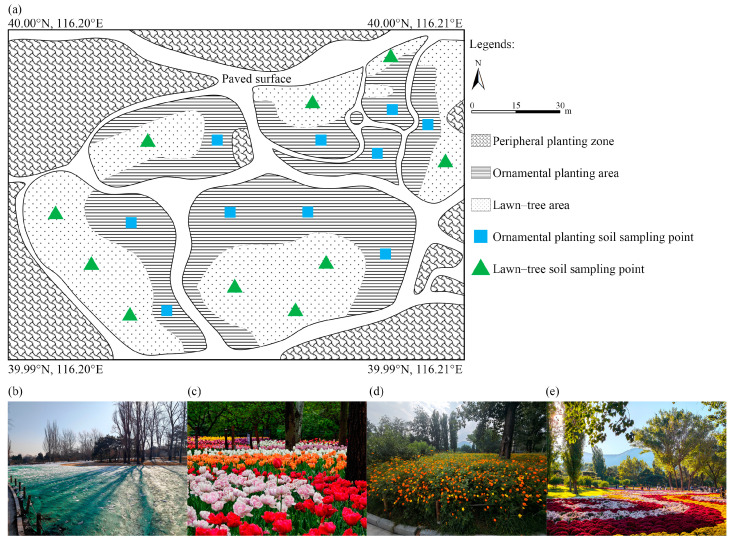
Overview of the experimental site and successive ornamental planting stages. (**a**) Layout of the ornamental planting area and adjacent lawn-tree reference area; (**b**) tulip bulb planting stage; (**c**) tulip flowering stage; (**d**) summer annual ornamental stage; (**e**) chrysanthemum flowering stage.

The study was conducted in the China National Botanical Garden (Northern Garden), Beijing, China (116.2043° E, 39.9953° N) (Figure 1a). The region is characterized by a temperate monsoon climate, with a mean annual temperature of approximately 11.6 °C and mean annual precipitation of 634.2 mm, concentrated in summer months [16].

The experimental site covers approximately 8000 m^2^ and comprises two adjacent systems: an intensively managed ornamental planting area (~5500 m^2^) and a comparatively low-disturbance lawn-tree area (~2500 m^2^). The native soil in this area is classified as Cinnamon soil according to the Chinese Soil Taxonomy [17]. However, long-term intensive horticultural management has substantially altered its physicochemical properties and is likely to have modified its morphological characteristics. Under the framework of the World Reference Base for Soil Resources (WRB), this soil corresponds to a Technic Technosol due to long-term accumulation of technogenic materials from intensive horticultural management (e.g., fertilizers, organic amendments, and physical disturbance) [18]. Consequently, although both systems share the same original soil parent material, they have likely undergone divergent pedogenic trajectories due to contrasting management histories. Therefore, for the purpose of this study, they are treated as distinct anthropogenically differentiated soil units rather than a single homogeneous soil entity.

The ornamental area has been subjected to continuous high-intensity horticultural management for over 22 years [19], including repeated tillage, seasonal plant replacement, fertilizer application, and irrigation, making it representative of ornamental soils under long-term disturbance in a botanical garden setting [11]. Although located within the Beijing metropolitan area, this botanical garden site represents a specific class of intensively managed ornamental soils under long-term horticultural rotation, which may differ from typical urban soils (e.g., park lawns, roadside soils, or community gardens) in terms of management intensity and disturbance regimes.

The ornamental planting area follows a structured annual multiple-cropping regime. Each November, after mechanical tillage and application of insecticide (dinotefuran) and fungicide (chlorothalonil), bulbous ornamentals—including *Tulipa* × *gesneriana* L., *Muscari botryoides* (L.) Mill., and *Hyacinthus orientalis* L.—are planted at a total density of approximately 205,000 bulbs, with tulips comprising over 97% of the planting (Figure 1b,c). Following the flowering period in May, bulbs are removed, and the soil is tilled prior to sowing summer annual ornamentals such as *Zinnia elegans* Jacq., *Helianthus annuus* L., and *Cosmos sulphureus* Cav. (~200,000 individuals) (Figure 1d). These are cleared in late August, after which soils undergo a second tillage event. In September, approximately 20,000 individuals of *Chrysanthemum* × *morifolium* (Ramat.) Hemsl. are transplanted and subsequently removed in November, initiating the next annual cycle (Figure 1e).

Spontaneous vegetation within the ornamental area includes *Chenopodium album* L., *Setaria viridis* (L.) P. Beauv., and *Taraxacum mongolicum* Hand.-Mazz. The adjacent lawn-tree area, used as a low-disturbance reference system, is dominated by woody perennials such as *Prunus* ‘Baihua Shanbitao’, *Populus nigra* var. *italica* (Moench) Koehne, *Sambucus nigra* L., and *Pinus tabuliformis* Carrière. Compared with the ornamental planting area, this reference system experiences substantially lower disturbance frequency and minimal seasonal plant turnover, allowing evaluation of management intensity effects under similar climatic and edaphic conditions.

### 2.2. Soil Sampling

Soil samples were collected immediately after the peak flowering stage of each ornamental planting cycle to capture representative microbial community states under active plant–soil interaction. Sampling was conducted simultaneously in both the ornamental planting area (high disturbance) and the adjacent lawn-tree area (low disturbance) to minimize seasonal bias. Ten sampling points were established in each zone (Figure 1a). At each point, soils were collected from a depth of 5–20 cm using a sterilized soil corer, targeting the active root zone while minimizing surface litter interference [11]. Three subsamples were randomly collected within a 1 m radius and homogenized to form one composite sample per point. Fresh soils were divided into two subsamples: one portion was stored at −80 °C for DNA extraction, and the other was air-dried for physicochemical analyses and enzyme activity determination.

### 2.3. Soil Physicochemical Properties and Enzyme Activities

Soil physicochemical properties and enzyme activities were analyzed by Nanjing Cavendish Testing Technology Co., Ltd. (Nanjing, China). Standard analytical procedures were followed as described by Bao [20]. Soil pH was determined using a potentiometric method. Soil organic carbon (SOC) was measured using the potassium dichromate oxidation method with external heating as described by Walkley and Black [21]. Total nitrogen (TN) was quantified using a Dumas combustion analyzer, while alkaline hydrolyzable nitrogen (AN) was determined using the alkaline diffusion method. Total phosphorus (TP) was measured using the alkali fusion–molybdenum antimony colorimetric method, and available phosphorus (AP) was extracted with NaHCO_3_ and quantified colorimetrically. Total potassium (TK) was determined by alkali fusion followed by flame photometry, and available potassium (AK) was extracted with ammonium acetate and measured by flame photometry.

Concentrations of soil heavy metals, including cadmium (Cd), cobalt (Co), copper (Cu), chromium (Cr), manganese (Mn), nickel (Ni), lead (Pb), zinc (Zn), vanadium (V), arsenic (As), molybdenum (Mo), antimony (Sb), and mercury (Hg), were determined using inductively coupled plasma mass spectrometry (ICP-MS) following acid digestion [22]. Soil enzyme activities were determined to characterize microbial functional potential. β-1,4-glucosidase (BG) and acid phosphatase (ACP) activities were measured using the p-nitrophenol colorimetric method. Sucrase (SUC) activity was determined using the 3,5-dinitrosalicylic acid method, urease (URE) activity was measured using the phenol–hypochlorite colorimetric method, and catalase (CAT) activity was determined by titration. Detailed protocols followed Bao (2000) [20] and Sinsabaugh et al. [23].

For all physicochemical and enzymatic analyses, each composite soil sample (*n* = 10 per treatment group) was measured in duplicate (technical replicates), and the average value was used for statistical analysis. If the coefficient of variation between the two replicates exceeded 5%, a third measurement was performed, and the median value was retained.

Redundancy analysis (RDA) was conducted in R (version 4.3.0) using the vegan package after Hellinger transformation of community matrices. Multicollinearity among environmental variables was assessed using variance inflation factors (VIFs), and only variables with VIFs < 10 were retained [24].

### 2.4. DNA Extraction, PCR Amplification, and Library Preparation

Total genomic DNA was extracted from soil samples using the DNeasy PowerSoil Kit (QIAGEN, Hilden, Germany) according to the manufacturer’s instructions. DNA extraction was performed once per composite soil sample (*n* = 10 per treatment group). Bacterial communities were characterized by amplifying the V4 hypervariable region of the 16S rRNA gene using primers 515F (5′-GTGCCAGCMGCCGCGGTAA-3′) and 806R (5′-GGACTACHVGGGTWTCTAAT-3′) [25]. Fungal communities were assessed by amplifying the internal transcribed spacer (ITS) region using primers ITS3F (5′-GCATCGATGAAGAACGCAGC-3′) and ITS4R (5′-TCCTCCGCTTATTGATATGC-3′) [26].

To ensure amplification accuracy, we performed PCR amplification using Phusion^®^ High-Fidelity PCR Master Mix with GC Buffer (New England Biolabs, Ipswich, MA, USA) to ensure amplification accuracy. Each reaction was prepared by mixing 12.5 μL of 2× Master Mix, 1 μL of each primer (10 μM stock), 10 ng of template DNA, and nuclease-free water to a final volume of 25 μL. The PCR program consisted of an initial denaturation at 98 °C for 1 min, followed by 30 cycles of denaturation at 98 °C for 10 s, annealing at 50 °C for 30 s, and extension at 72 °C for 30 s, with a final extension at 72 °C for 5 min [27]. To minimize stochastic amplification bias, PCR amplification was performed in triplicate per DNA sample (technical replicates). The three replicate products were pooled prior to purification.

The resulting PCR products were purified using the GeneJET^TM^ Gel Extraction Kit (Thermo Scientific, Waltham, MA, USA). Sequencing libraries were constructed using the TruSeq^®^ DNA PCR-Free Sample Preparation Kit, and index adapters were added according to the manufacturer’s protocol. Library quality and concentration were assessed using a Qubit 2.0 Fluorometer (Thermo Scientific, Waltham, MA, USA) and an Agilent 2100 Bioanalyzer (Agilent, Santa Clara, CA, USA). Each library was sequenced once on an Illumina MiSeq PE250 platform at Novogene Co., Ltd. (Beijing, China) [5], generating paired-end reads (2 × 250 bp).

### 2.5. Bioinformatics Processing and Taxonomic Assignment

Raw paired-end sequencing reads were processed using QIIME2 (version 2024.10). Demultiplexed sequences were quality-filtered, denoised, merged, and chimera-checked using the DADA2 plugin implemented within QIIME2, generating high-resolution amplicon sequence variants (ASVs) [28]. Taxonomic classification of bacterial ASVs was performed using a naïve Bayesian classifier trained against the SILVA v138.2 reference database (https://www.arb-silva.de/, accessed on 8 February 2026) [29], while fungal ASVs were assigned using the UNITE v10.0 database (https://unite.ut.ee/, accessed on 8 December 2025) [30]. Prior to downstream analyses, we removed ASVs annotated as chloroplasts, mitochondria, host-derived sequences, or unassigned at the kingdom level. To reduce the influence of rare taxa and potential sequencing artifacts, ASVs were retained for downstream analyses only if they were present in at least 20% of all samples [31]. Community matrices were rarefied to an equal sequencing depth prior to diversity analyses to minimize bias associated with uneven sequencing effort.

Alpha diversity indices, including Chao1 richness, Shannon diversity, and Simpson diversity, were calculated using the vegan package in R. Differences among groups were evaluated using the Kruskal–Wallis test. When significant effects were detected, pairwise comparisons were performed using Dunn’s post hoc test with Benjamini–Hochberg (BH) false discovery rate correction [32]. Beta diversity was assessed using Bray–Curtis dissimilarity metrics and visualized by principal coordinate analysis (PCoA). Permutational multivariate analysis of variance (PERMANOVA) was conducted using the adonis function in the vegan package to evaluate the effects of planting stage and site type on community composition [33].

Microbial co-occurrence networks were constructed separately for bacterial and fungal communities. Prior to network construction, ASVs with relative abundance < 0.01% or present in fewer than 50% of samples were removed to reduce spurious correlations driven by rare taxa. Spearman rank correlations were calculated among retained ASVs, and *p*-values were adjusted using the BH method. Only robust correlations (|r| > 0.6 and adjusted *p* < 0.01) were included in network construction [34]. Network topological properties, including number of nodes, number of edges, average degree, graph density, modularity, and average path length, were calculated using the igraph package in R. Network visualization was performed using Gephi v0.10.1.

## 3. Results

### 3.1. Planting-Driven Shifts in Soil Properties and Enzyme Activities

Soil physicochemical properties and enzyme activities differed markedly between the intensively managed ornamental planting area (O) and the adjacent lawn-tree area (L) across successive ornamental planting stages (Table 1). At the tulip stage (L1 vs. O1), 20 of the 26 measured variables showed significant differences between sites. Similar patterns were observed during the summer annual (L2 vs. O2) and chrysanthemum (L3 vs. O3) stages, with 19 variables differing significantly at each stage. Only a small subset of heavy metals (e.g., Pb, V, As, Sb, and Co, depending on stage) and selected enzyme activities (e.g., BG or URE) showed no consistent site-level differences.

Across all planting stages, the ornamental planting area generally exhibited significantly higher concentrations of key nutrient indicators, including SOC, TN, AN, TP, AP, and AK, compared with the lawn-tree area. In contrast, heavy metal concentrations displayed less consistent site-dependent patterns, with some elements varying by stage but without a uniform directional trend.

Within-site comparisons revealed distinct temporal dynamics. In the lawn-tree area (L1–L3), nearly all measured variables differed significantly across stages, indicating pronounced seasonal or vegetation-associated variation. In contrast, within the ornamental planting area (O1–O3), five heavy metals (Co, Mn, Ni, Pb, and Hg) showed no significant stage-dependent variation, whereas most nutrient-related variables and enzyme activities changed significantly across successive planting stages.

Collectively, these results indicate that intensive ornamental management consistently altered soil nutrient pools relative to the low-disturbance reference system, while temporal variability was more pronounced for nutrient and enzyme parameters than for heavy metal concentrations.

### 3.2. Alpha-Diversity Responses to Successive Ornamental Planting Cycles

**Figure 2:**
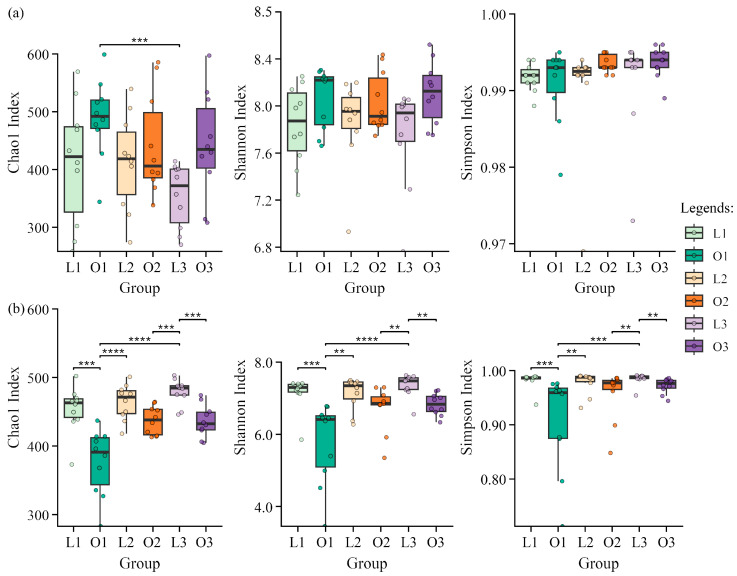
Alpha-diversity indices of soil bacterial (**a**) and fungal (**b**) communities in the ornamental planting area (O) and adjacent lawn-tree area (L) across successive ornamental planting stages. Alpha diversity was estimated using Chao1, Shannon, and Simpson indices. Boxes represent the interquartile range (IQR), center lines indicate medians, whiskers denote 1.5 × IQR, and points represent individual samples. Asterisks indicate significant differences between sites within the same planting stage (** *p* < 0.01, *** *p* < 0.001, **** *p* < 0.0001; Kruskal–Wallis test followed by Dunn’s post hoc test with BH correction). Planting stages: tulip (L1/O1), summer annual ornamentals (L2/O2), chrysanthemum (L3/O3).

Alpha-diversity patterns of soil bacterial and fungal communities differed in their responses to site type and successive ornamental planting stages (Figure 2).

For bacterial communities (Figure 2a), richness (Chao1) and diversity indices (Shannon and Simpson) showed limited variation across planting stages in both site types. No significant differences were detected between the ornamental planting area (O) and the lawn-tree area (L) within any planting stage (*p* > 0.05). Although bacterial diversity indices were consistently slightly higher in the ornamental planting area than in the lawn-tree area, these differences were not statistically significant. Similarly, within-site comparisons across stages revealed no significant temporal shifts in bacterial alpha diversity.

In contrast, fungal communities exhibited pronounced site-dependent and stage-dependent variation (Figure 2b). The lawn-tree area generally maintained higher fungal richness and diversity compared with the ornamental planting area. Significant site-level differences were observed during the tulip stage (L1 vs. O1) and the chrysanthemum stage (L3 vs. O3) (*p* < 0.05). Notably, fungal diversity in O1 was lower than in other planting-stage groups. In addition, within-site comparisons indicated significant variation in fungal alpha diversity across planting stages, particularly within the ornamental planting area.

Overall, bacterial alpha diversity remained relatively stable across both site types and planting stages, whereas fungal alpha diversity showed stronger spatial and temporal variability.

### 3.3. Planting-Stage Shifts in Bacterial and Fungal Community Composition

**Figure 3:**
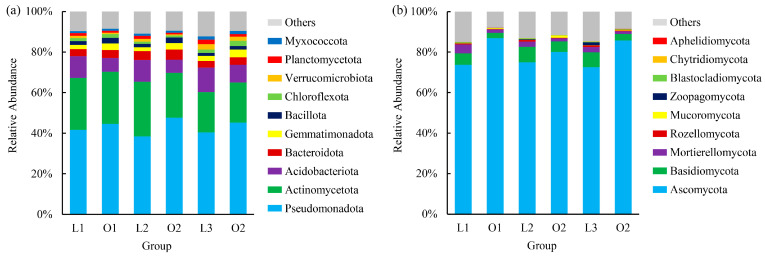
Relative abundances of dominant bacterial (**a**) and fungal (**b**) phyla across site types and successive planting stages. “O” denotes the ornamental planting area, and “L” denotes the adjacent lawn-tree area. Planting stages include tulip (L1/O1), summer annual ornamentals (L2/O2), and chrysanthemum (L3/O3). “Others” represents phyla with low relative abundance (<1%) or unclassified taxa.

Across both site types and planting stages, a total of 12 bacterial and 9 fungal phyla were detected. Among bacteria, ten phyla each exceeded 1% relative abundance (Figure 3a, Table S1), whereas fungal communities were largely dominated by only three phyla (Figure 3b, Table S2).

Bacterial communities were consistently dominated by Pseudomonadota (43.04% on average), followed by Actinomycetota (23.29%) and Acidobacteriota (9.25%) (Figure 3a). Together, these three phyla accounted for the majority of sequences across all sites and planting stages, indicating a relatively stable core bacterial assemblage.

Site-level comparisons within individual planting stages revealed moderate compositional differences in bacterial communities. During the tulip stage (L1 vs. O1), significant differences were observed in Acidobacteriota and Gemmatimonadota. A broader set of phyla differed during the summer annual stage (L2 vs. O2), including Pseudomonadota, Acidobacteriota, Gemmatimonadota, Bacillota, and Planctomycetota. After the chrysanthemum stage (L3 vs. O3), significant differences were limited to Gemmatimonadota, Chloroflexota, and Planctomycetota (Figure 3a, Table S1). Within-site comparisons across planting stages indicated temporal variation in several bacterial phyla; however, dominant phyla maintained relatively consistent proportions over time.

In contrast, fungal communities displayed a more uneven dominance structure. Ascomycota overwhelmingly dominated across samples (79.01% on average), followed by Basidiomycota (5.30%) and Mortierellomycota (2.33%) (Figure 3b). The remaining phyla contributed only minor fractions to total community composition.

Fungal communities exhibited more pronounced compositional differentiation between sites and planting stages. During the tulip stage (L1 vs. O1), significant differences were detected across multiple phyla, including Ascomycota, Basidiomycota, Mortierellomycota, Blastocladiomycota, and Chytridiomycota. Fewer differences were observed during the summer annual stage, whereas the chrysanthemum stage showed significant differences across nearly all major fungal phyla (Figure 3b, Table S2). Within-site temporal variation was comparatively limited, affecting only a small subset of phyla. Overall, fungal communities demonstrated greater compositional differentiation between site types and planting stages than bacterial communities.

### 3.4. Contrasting Responses of Bacterial and Fungal Communities to Site Type and Planting Stage

**Figure 4:**
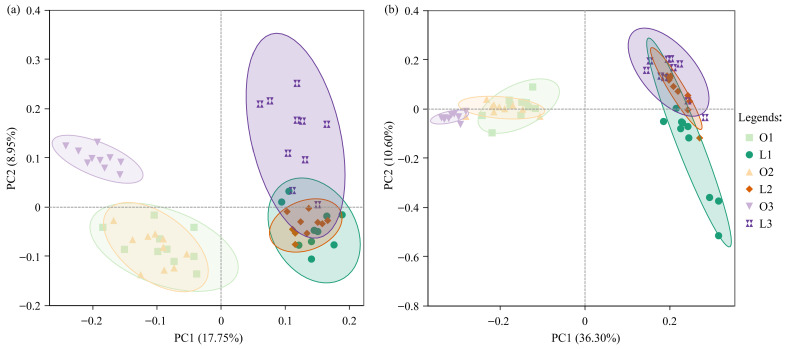
Principal coordinate analysis (PCoA) based on Bray–Curtis dissimilarity showing the effects of site type and planting stage on bacterial (**a**) and fungal (**b**) community composition. The percentages on the axes indicate the proportion of variation explained. Ellipses represent 95% confidence intervals around group centroids. L1 and O1: tulip stage (lawn-tree and ornamental areas, respectively); L2 and O2: summer annual stage; L3 and O3: chrysanthemum stage.

Principal coordinate analysis (PCoA) based on Bray–Curtis dissimilarity was performed to assess differences in bacterial and fungal community composition among site types and planting stages (Figure 4).

For bacterial communities, the first two PCoA axes explained 26.70% of the total variation (Figure 4a). Permutational multivariate analysis of variance (PERMANOVA) revealed that both site type (R^2^ = 0.168, *p* = 0.001) and planting stage (R^2^ = 0.180, *p* = 0.001) significantly influenced bacterial community composition, with similar explanatory power (Tables S3 and S4). The interaction between site type and planting stage was not significant (*p* = 0.392). Pairwise comparisons indicated that the chrysanthemum stage differed significantly from both the tulip and summer annual stages (*p* < 0.01), whereas no significant difference was detected between the tulip and summer annual stages (Table S5).

Permutational analysis of multivariate dispersions (PERMDISP) revealed significant heterogeneity in multivariate dispersions among planting stages (*p* = 0.004) (Tables S6 and S7), which should be considered when interpreting the PERMANOVA results. Nevertheless, the clear separation of group centroids in the PCoA ordination suggests that planting stage-related shifts in community composition contribute to the observed differences beyond dispersion effects.

For fungal communities, the first two PCoA axes explained 46.90% of the total variation (Figure 4b). Permutational multivariate analysis of variance (PERMANOVA) results indicated a significant effect of site type on fungal community composition (R^2^ = 0.225, *p* = 0.001), whereas planting stage had no significant effect (*p* = 0.461), and the interaction term was not significant (*p* = 0.058) (Tables S8 and S9). PERMDISP analysis showed no significant differences in dispersion among groups (*p* > 0.05) (Tables S10 and S11).

Collectively, these results indicate contrasting response patterns between bacterial and fungal communities: bacterial composition was influenced by both site type and planting stage, whereas fungal composition was primarily structured by site characteristics and exhibited limited temporal variation across planting stages.

### 3.5. Environmental Drivers of Microbial Community Composition

**Figure 5:**
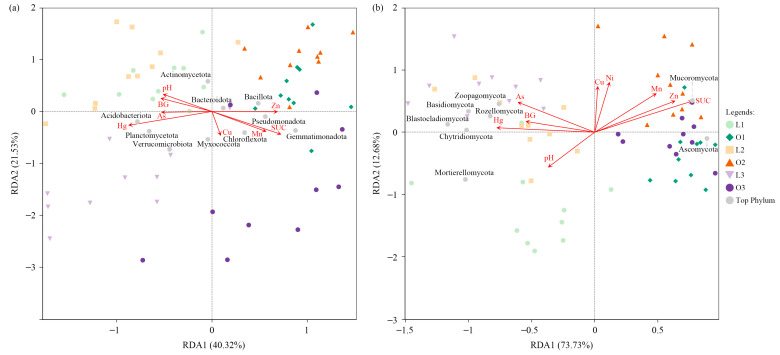
Redundancy analysis (RDA) illustrating relationships between soil environmental variables and microbial community composition for bacteria (**a**) and fungi (**b**). The percentages on the axes represent the proportion of variation explained. Arrows indicate environmental variables. L1 and O1: tulip stage (lawn-tree and ornamental areas, respectively); L2 and O2: summer annual stage; L3 and O3: chrysanthemum stage.

Redundancy analysis (RDA) was conducted to evaluate the relationships between soil physicochemical properties, enzyme activities, and microbial community composition (Figure 5, Tables S12 and S13).

For bacterial communities, the first two RDA axes explained 61.85% of the total variation (Figure 5a), indicating a strong association between environmental variables and community structure. Both soil enzyme activities and heavy metals were significantly correlated with bacterial composition. Among the measured variables, SUC (r^2^ = 0.5140, *p* < 0.001) and Hg (r^2^ = 0.4833, *p* < 0.001) exhibited the strongest explanatory power (Table S12). Sucrase (SUC) activity was positively associated with Gemmatimonadota, Pseudomonadota, and Bacillota, whereas it was negatively correlated with Acidobacteriota, Actinomycetota, and Planctomycetota (Figure S1a). In contrast, Hg displayed the opposite correlation pattern, being positively associated with Acidobacteriota, Planctomycetota, and Verrucomicrobiota, and negatively associated with Pseudomonadota, Gemmatimonadota, and Bacillota.

For fungal communities, environmental variables explained an even greater proportion of variation, with the first two RDA axes accounting for 86.41% of the total variation (Figure 5b). Similar to bacterial communities, enzyme activities and heavy metals were strongly associated with fungal composition. Sucrase (SUC) activity (r^2^ = 0.6760, *p* < 0.001) and Zn (r^2^ = 0.5144, *p* < 0.001) were identified as the most influential predictors (Table S13). Zinc (Zn) showed a significant negative correlation with Chytridiomycota (Figure S1b). Overall, environmental variables exhibited strong associations with both bacterial and fungal communities, with fungal composition showing a higher proportion of explained variation than bacterial composition.

### 3.6. Network Complexity and Organization of Soil Microbial Communities

**Figure 6:**
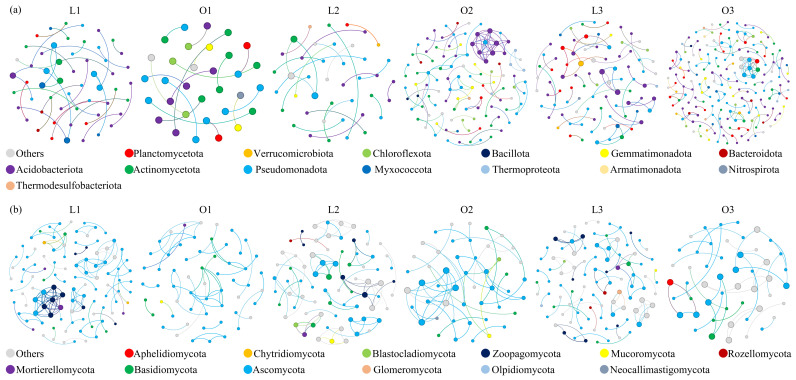
Co-occurrence networks of soil bacterial (**a**) and fungal (**b**) communities across site types and planting stages. Node size is proportional to degree (number of connections), and node colors represent taxonomic affiliation at the phylum level. Positive correlations are shown as solid lines and negative correlations as dashed lines. L1 and O1: tulip stage (lawn-tree and ornamental areas, respectively); L2 and O2: summer annual stage; L3 and O3: chrysanthemum stage.

Co-occurrence network analysis revealed pronounced differences in microbial network structure across site types and planting stages (Figure 6, Tables S14–S17).

For bacterial communities, network size and connectivity varied markedly between sites and across planting stages. During the tulip stage (L1 vs. O1), the lawn-tree area exhibited higher numbers of nodes and edges than the ornamental planting area. However, during the summer annual and chrysanthemum stages (L2 vs. O2 and L3 vs. O3), the ornamental planting area displayed larger and more connected networks (Figure 6, Table S14). Within the lawn-tree area, network size decreased from L1 to L2 and subsequently increased at L3, whereas in the ornamental planting area, network size increased progressively across planting stages.

Fungal networks exhibited a contrasting pattern. Across all planting stages, the lawn-tree area consistently maintained higher node and edge numbers than the ornamental planting area (Figure 6, Table S15). Both site types showed a decrease followed by an increase in network size across planting stages, indicating similar temporal dynamics in fungal network organization.

In both bacterial and fungal networks, positive correlations substantially outnumbered negative correlations regardless of site or planting stage, indicating that co-occurrence relationships were predominantly positive (Figure 6, Tables S14 and S15). At the phylum level, bacterial network nodes were primarily affiliated with Acidobacteriota, Actinomycetota, and Pseudomonadota across most groups, whereas the L3 bacterial network showed increased representation of Planctomycetota (Table S16). Fungal networks were consistently dominated by Ascomycota, which accounted for 34–69% of network nodes across all groups (Table S17).

Taken together, bacterial networks displayed stronger site- and stage-dependent restructuring, whereas fungal networks exhibited more consistent site-level differences with comparatively stable taxonomic dominance patterns.

## 4. Discussion

### 4.1. Intensive Ornamental Planting Reshapes Botanical Garden Cinnamon Soil Biogeochemistry

Intensive ornamental planting substantially reshaped the physicochemical environment of soils, resulting in clear differences between the ornamental planting area and the adjacent lawn-tree area. Soil properties and enzyme activities are jointly influenced by terrain, soil type, climate, vegetation, and land management, leading to pronounced spatial heterogeneity [35]. Among these factors, soil organic carbon (SOC) plays a central role, as it directly reflects soil fertility and regulates nutrient availability and microbial enzymatic activity [36]. In the present study, the distribution patterns of SOC were closely aligned with those of TN, AN, TP, AP, and AK across sites and planting stages, confirming the integrative role of SOC in structuring soil fertility gradients.

Nitrogen (N), phosphorus (P), and potassium (K) are fundamental nutrients regulating plant growth, biomass allocation, and soil microbial activity [37,38]. Their concentrations therefore have cascading effects on microbial biomass and enzyme production [39]. In this system, tulip cultivation represented the longest planting period and imposed the highest nutrient demand. During bulb development, tulips rely not only on stored reserves but also absorb substantial external nutrients, with reported uptake of approximately 180 mg N, 33 mg P_2_O_5_ (phosphorus pentoxide), and 50 mg K_2_O (potassium oxide) per plant [40,41]. Despite this high demand, available nutrients remained relatively abundant after the chrysanthemum stage, indicating that cumulative fertilization and management inputs exceeded plant nutrient consumption and generated residual fertility. This suggests that current nutrient levels could potentially sustain subsequent planting cycles without additional fertilization.

Across all planting stages, the ornamental planting area exhibited significantly higher SOC, TN, AN, TP, and AP than the lawn-tree area. Such enrichment likely reflects more intensive fertilization, irrigation, and biomass turnover associated with ornamental management practices. Increased organic inputs and faster decomposition of plant residues may further contribute to SOC accumulation in the ornamental plots. These findings support previous observations that land-use intensity can substantially alter soil background values and nutrient dynamics [42].

Soil enzyme activities showed stage-dependent responses that mirrored shifts in nutrient availability and disturbance intensity. β-glucosidase (BG), a key enzyme in cellulose decomposition and carbon cycling, generally exhibited positive associations with SOC, suggesting that carbon availability stimulates microbial investment in cellulose-degrading enzymes [42]. However, after the chrysanthemum stage, BG activity was significantly lower in the ornamental area despite its higher SOC. This apparent decoupling may reflect mechanical disturbance (e.g., tillage and litter removal) in the ornamental plots, which disrupts cellulose accumulation and microbial-substrate continuity. In contrast, litter accumulation in the lawn-tree area may have enhanced cellulose inputs and stimulated BG activity.

Acid phosphatase (ACP), a key enzyme involved in organic P mineralization, typically increases under P-limited conditions [43]. Interestingly, ACP activity was consistently higher in the ornamental planting area across all stages despite higher background P levels. This may indicate strong plant-driven P demand from tulip and chrysanthemum cultivation, which stimulates microbial and root-associated phosphatase production. Alternatively, elevated organic P pools in fertilized soils may require sustained enzymatic mineralization [39].

Sucrase (SUC) activity, which hydrolyzes sucrose into readily available carbon sources, increased across planting stages, likely reflecting enhanced root exudation and rhizosphere activity during plant growth. This pattern highlights the rapid physiological responsiveness of soil microbial communities to changing carbon inputs, consistent with recent findings emphasizing microbial functional plasticity under dynamic plant–soil interactions [44].

Urease (URE) activity, which catalyzes urea hydrolysis and contributes to nitrogen mineralization, declined after the chrysanthemum stage in both areas. This decrease may be associated with reduced plant nitrogen uptake and diminished rhizosphere stimulation during late-season senescence [45]. In contrast, higher URE activity during the tulip stage likely reflected elevated organic nitrogen availability and intensified microbial nitrogen turnover [43].

Catalase (CAT) activity was significantly higher in the lawn-tree area than in the ornamental planting area. As CAT activity decomposes hydrogen peroxide and reflects oxidative stress regulation, its higher activity may indicate more active aerobic microbial metabolism in structurally stable and well-aerated soils. Dense grass roots and deep tree rooting systems may improve soil structure and oxygen diffusion, thereby enhancing oxidative metabolic processes [35]. These results suggest that soil physical properties may exert stronger control over CAT activity than organic matter concentration alone.

Heavy metal dynamics further revealed the imprint of intensive ornamental management. Most metals (e.g., Cu, Cr, Ni, Pb, Zn, V, Mo) were generally higher in the ornamental planting area, likely reflecting fertilizer and pesticide inputs, which are recognized anthropogenic sources of Cu and Zn [46]. In contrast, some metals (e.g., As, Hg, Pb) peaked in the lawn-tree area after the chrysanthemum stage, possibly due to atmospheric deposition and seasonal litter return [22]. Plant tissues can accumulate heavy metals from deeper soil layers and subsequently enrich surface soils through litterfall [47]. In addition, tillage, transplanting, and wet–dry cycles may alter redox conditions and influence the mobility and redistribution of metal fractions.

Notably, concentrations of Cr, Cu, Hg, Ni, and Zn exceeded reported average levels in Chinese agricultural soils [48]. According to geoaccumulation index calculations based on urban soil background values in Beijing [49], Hg reached a moderate pollution level, while Cd, Cu, Mo, Ni, and Zn showed slight pollution, and other metals remained within clean categories. These findings indicate that ornamental planting systems in botanical gardens may experience cumulative heavy metal inputs that warrant long-term ecological monitoring.

Overall, intensive ornamental planting reshapes soil biogeochemistry through nutrient enrichment, enzymatic adjustment, disturbance-driven substrate redistribution, and anthropogenic metal inputs. These interacting processes generate dynamic soil environments that provide the foundation for subsequent shifts in microbial diversity and community organization.

### 4.2. Divergent Reassembly of Bacterial and Fungal Communities Driven by Carbon Availability and Heavy Metal Stress

The RDA results revealed that soil enzyme activities and heavy metals jointly shaped both bacterial and fungal community composition, albeit with distinct dominant drivers and response patterns. For bacterial communities, SUC activity and Hg concentration emerged as the strongest predictors, collectively explaining a substantial portion of community variation. The positive correlation between SUC activity and copiotrophic phyla (e.g., Pseudomonadota, Gemmatimonadota, Bacillota) aligns with their well-documented preference for labile carbon inputs, such as root exudates and fresh organic matter [13]. Conversely, the negative correlation of SUC activity with oligotrophic Acidobacteriota and Actinomycetota reflects their adaptive strategy to resource-limited niches, where they rely on complex organic compounds and exhibit slower growth rates [50,51]. This divergent response to carbon availability underscores the ecological differentiation among bacterial phyla along the resource spectrum.

The strong association of Hg with bacterial community composition, particularly its positive correlation with Acidobacteriota, Planctomycetota, and Verrucomicrobiota, and negative correlation with Pseudomonadota and Bacillota, suggests that heavy metal contamination acts as a selective filter favoring taxa with inherent or adaptive metal tolerance mechanisms [52]. Planctomycetota, for instance, have been reported to possess diverse metal resistance genes and thrive in contaminated environments, potentially due to their unique cell wall structure and metabolic versatility [53].

For fungal communities, the even higher explanatory power of the RDA model (86.41%) indicates that fungal assembly is more tightly constrained by the measured environmental variables. Sucrase (SUC) activity remained a dominant factor, reinforcing the central role of carbon availability in structuring both bacterial and fungal communities [54]. The significant negative correlation between Zn and Chytridiomycota is noteworthy, as this phylum comprises early-diverging fungi that are often associated with aquatic or moist habitats and sensitive to metal stress [55]. The contrasting responses of bacteria and fungi to heavy metals—Hg for bacteria versus Zn for fungi—may reflect phylum-specific physiological sensitivities or differential accumulation patterns of these metals in soil microhabitats [55]. Collectively, these findings demonstrate that intensive ornamental management simultaneously modulates microbial communities through nutrient enrichment and metal-induced environmental filtering, with the relative importance of each factor varying across microbial kingdoms.

### 4.3. Taxon-Specific Responses Reveal Contrasting Reassembly Pathways of Bacterial and Fungal Communities

Pseudomonadota represented the most abundant bacterial phylum (mean relative abundance 43.04%). As one of the metabolically most versatile bacterial groups, members of this phylum are typically copiotrophic and respond rapidly to nutrient enrichment and rhizosphere carbon inputs [13]. The consistently higher SUC activity in the ornamental planting area aligns with the copiotrophic strategy of Pseudomonadota, which efficiently utilizes labile carbon sources derived from root exudates. Their dominance, therefore, reflects frequent fertilization and dynamic carbon fluxes associated with ornamental planting. Actinomycetota (23.29%) represented the second most abundant phylum. Although generally considered slower-growing, many members possess strong stress tolerance, sporulation capacity, and the ability to degrade complex organic substrates [50]. The gradual decline of Actinomycetota across planting stages may indicate sensitivity to repeated disturbance or shifts in soil aeration conditions. As obligate aerobes with active respiratory metabolism [56], their ecological success is closely tied to oxidative processes. However, increasing CAT activity across stages suggests enhanced oxidative detoxification capacity in soil, potentially reducing selective pressure favoring Actinomycetota dominance. Acidobacteriota (9.25%), typically considered oligotrophic and competitive under low-nutrient conditions [51], displayed patterns consistent with environmental filtering. Although often associated with acidic soils, certain subgroups (e.g., GP4, GP6) are well adapted to neutral to alkaline conditions. Their distribution between sites likely reflects fine-scale nutrient gradients rather than bulk pH alone. The presence of specific subgroups under alkaline conditions suggests local adaptation within this phylum.

Together, these patterns indicate that bacterial community composition is tightly linked to short-term nutrient pulses, enzymatic activity, and disturbance intensity. Frequent tillage, fertilization, and plant turnover likely create fluctuating microhabitats that favor metabolically flexible and opportunistic taxa. This explains why bacterial beta diversity was strongly influenced by both planting stage and site type.

In contrast to bacteria, fungal communities were overwhelmingly dominated by Ascomycota (79.01%), the largest and ecologically most versatile fungal phylum [57]. Their dominance suggests active organic matter turnover and strong saprotrophic activity. Given their capacity to produce extracellular enzymes, Ascomycota likely contribute substantially to observed BG and SUC activities [26]. Basidiomycota (5.30%) were less abundant in the ornamental planting area, likely due to the limited availability of ectomycorrhizal host plants such as woody species [58]. This distribution pattern reflects habitat filtering driven by vegetation structure rather than short-term nutrient changes. Mortierellomycota (2.33%), often associated with nitrogen-rich environments [59], showed patterns potentially constrained by disturbance intensity, as members of this phylum are reported to decline under frequent tillage or chemical application [60].

Unlike bacteria, fungal community composition showed limited sensitivity to planting stage. This relative stability can be explained by fungal life-history traits. Filamentous growth and hyphal networks enable fungi to explore larger soil volumes and redistribute nutrients internally [61], reducing dependence on localized resource pulses. Consequently, fungal communities appear more strongly structured by persistent site characteristics such as soil structure, root architecture, and long-term organic matter inputs. This divergence suggests that fungal reassembly encodes longer-term habitat imprinting, whereas bacterial reassembly responds to transient management signals [62]. Such differentiation provides a mechanistic explanation for why the planting stage significantly influenced bacterial beta diversity but not fungal composition.

Given that tulip and chrysanthemum are highly susceptible to soil-borne fungal pathogens—including *Fusarium* spp., *Botrytis* spp., and *Puccinia* spp. [63,64]—soil health represents a critical management concern. Notably, only *Fusarium solani* was detected, and at an extremely low relative abundance. Moreover, no visible disease symptoms were observed in the field. Higher soil enzyme activities (ACP, BG, SUC) may indicate active microbial communities capable of suppressing pathogen establishment through competitive exclusion and nutrient competition [5]. These observations suggest that, despite intensive planting, the current soil microbial environment remains functionally resilient. However, tulip and chrysanthemum are well recognized as continuous-cropping-sensitive crops, meaning that repeated cultivation in the same soil can exacerbate soil-borne disease pressure and microbial imbalance [65,66,67]. Because soil-borne pathogens can accumulate progressively under continuous planting [68], long-term monitoring of soil physicochemical properties and microbial community dynamics is essential to maintain planting sustainability.

Collectively, these findings demonstrate that intensive ornamental planting does not uniformly restructure the soil microbiome. Instead, it selectively amplifies nutrient-responsive bacterial taxa while fungal assemblages remain constrained by site-level legacy effects. Recognizing this taxon-specific assembly divergence is essential for understanding how short-term management interacts with long-term soil ecological stability in such intensively managed systems.

### 4.4. Intensive Ornamental Planting Restructures Microbial Interaction Networks and Alters Stability Trajectories

Bacterial co-occurrence networks exhibited pronounced reassembly across planting stages, particularly in the ornamental planting area. While the lawn-tree area initially showed higher connectivity at the early stage (L1 > O1 in node number, edge number, and average degree), the pattern reversed during subsequent stages, with O2 and O3 consistently exceeding their lawn counterparts in network size and connectivity. This progressive increase in nodes, edges, positive associations, and average degree from O1 to O3 indicates that intensive ornamental planting promotes cumulative interaction complexity. Such a pattern suggests repeated fertilization, irrigation, and tillage create fluctuating yet resource-rich microhabitats that stimulate metabolic interdependence among taxa [7]. Importantly, negative edges and modularity were consistently higher in ornamental soils across all stages. An increase in negative associations typically signifies stronger niche competition and environmental filtering, potentially stabilizing the community via competitive balance [69]. Higher modularity further suggests partitioned interactions, which limit the spread of disturbances across the network [70]. Together, these features suggest that ornamental management drives bacterial communities toward a more interactive yet structurally compartmentalized configuration.

Graph density decreased progressively from O1 to O3, despite increasing node and edge numbers. This indicates that while the network expands, connections become more selectively organized rather than randomly intensified. In contrast, bacterial networks in the lawn-tree area followed a contraction–recovery pattern (decline at L2, recovery at L3), consistent with seasonal but less management-driven environmental fluctuation. This divergence strongly supports the hypothesis that bacterial networks encode short-term management signals [14].

Fungal networks exhibited a fundamentally different response pattern. Across all planting stages, node number, edge number, positive associations, and average degree were consistently higher in the lawn-tree area than in ornamental soils. In ornamental soils, edge number and positive associations declined steadily from O1 to O3, suggesting progressive simplification of fungal interaction webs. However, modularity displayed a recovery at O3, indicating strengthened compartmentalization despite reduced connectivity. Such decoupling between connectivity and modularity suggests that fungal communities may reorganize internally rather than expand interaction breadth under disturbance. This supports the ecological theory that fungal hyphal networks allow spatial resource translocation, buffering against short-term environmental pulses [71]. Thus, fungal networks appear more strongly structured by persistent habitat characteristics than by planting stage.

Across all bacterial networks, keystone taxa were consistently dominated by three major phyla: Pseudomonadota, Acidobacteriota, and Actinomycetota. Notably, their proportional contributions shifted subtly across stages, but no novel phylum emerged as a dominant keystone under ornamental management. This indicates that interaction architecture reorganization occurred primarily through rewiring among persistent dominant groups rather than taxonomic replacement [72]. In the lawn-tree late stage (L3), Planctomycetota emerged among keystone taxa, potentially reflecting increased organic matter stabilization and nitrogen cycling complexity [53]. Fungal keystone taxa were overwhelmingly dominated by Ascomycota, accounting for 34–69% of keystone nodes across sites and stages. Secondary contributors included Basidiomycota and low-abundance groups such as Mortierellomycota. The dominance of Ascomycota keystones suggests that saprotrophic decomposition remains the central organizing axis of fungal interaction networks in this system [26]. Crucially, keystone taxa were not necessarily the most abundant taxa, reinforcing the principle that ecological influence does not scale linearly with relative abundance [73].

Integrating metrics of connectivity, modularity, and the proportion of negative edges reveals contrasting stability trajectories between bacterial and fungal networks. In ornamental soils, bacterial networks became increasingly complex and modular, suggesting enhanced metabolic activity coupled with competitive structuring [7]. Fungal networks, by contrast, displayed reduced connectivity while preserving or recovering modularity, indicating structural buffering rather than expansion [15]. These patterns suggest that intensive ornamental planting promotes a dynamic–modular bacterial network and a conservative, compartmentalized fungal network. In ecological terms, bacteria respond rapidly to short-term resource pulses and disturbance events [14], whereas fungi encode longer-term habitat filtering and structural memory [54]. At the network level, therefore, bacterial communities capture the intensity of management practices, while fungal networks reflect site legacy [74,75].

Integrating these observations, we propose a dual-speed reassembly framework in which bacterial networks act as fast-responding systems with dynamic interaction networks that track short-term environmental fluctuations, whereas fungal networks function as slow-buffering, structurally persistent systems that maintain long-term stability. This divergence suggests that microbial communities achieve ecosystem resilience through complementary strategies: rapid bacterial responsiveness enables functional adjustment, while fungal structural stability preserves network integrity. Bacterial taxa, particularly copiotrophic groups such as Pseudomonadota, are characterized by rapid growth, metabolic flexibility, and strong responsiveness to resource pulses, enabling them to act as early responders to management-induced environmental fluctuations [13,14]. In contrast, fungal communities in this system, overwhelmingly dominated by Ascomycota, exhibit traits consistent with stress tolerance and persistence. Their filamentous growth form facilitates spatial redistribution of resources, buffering localized disturbances, while diverse enzymatic capabilities and spore-based dispersal contribute to persistence under repeated soil disturbance [4,5,10].

From a network perspective, these contrasting strategies are reflected in differences in interaction organization. Bacterial networks tend to exhibit higher temporal variability and are more sensitive to short-term environmental changes, whereas fungal networks display greater structural persistence and are more strongly associated with stable habitat conditions [9]. The coexistence of these two ecological strategies—bacterial responsiveness and fungal buffering—may enhance the overall resilience of botanical garden soil systems under intensive management, enabling rapid functional adjustment without compromising long-term structural stability.

Intensive ornamental planting does not simply increase microbial activity; it reorganizes interaction architecture in a guild-specific manner—enhancing bacterial network dynamism while preserving fungal structural compartmentalization [72]. Such dual reassembly may decouple short-term functional responsiveness from long-term ecological stability within urban soils [76]. Collectively, these network-level reorganizations are driven by the interactive effects of soil nutrients, enzyme activities (particularly SUC activity), and heavy metals (e.g., Hg for bacteria, Zn for fungi), which collectively act as environmental filters that differentially shape bacterial and fungal reassembly trajectories.

### 4.5. Practical Implications for Botanical Garden Cinnamon Soil Management

Our findings offer several actionable insights for the management of intensively cultivated botanical garden soils. First, the fast-responsive nature of bacterial networks suggests that routine monitoring of soil enzyme activities—particularly SUC and BG activities—could serve as sensitive early indicators of management-induced soil functional changes [13,14]. These enzymatic parameters respond rapidly to nutrient pulses and disturbance, providing a practical tool for assessing soil biological status without exhaustive sequencing. Second, the structural persistence of fungal networks underscores the importance of minimizing physical disturbance to preserve hyphal connectivity. Practices such as reduced tillage, maintaining perennial vegetation buffers, and avoiding excessive soil compaction could help sustain fungal-mediated ecosystem functions (e.g., organic matter decomposition and pathogen suppression) [15,61]. Third, the accumulation of certain heavy metals (e.g., Hg, Cu, Zn) in ornamental soils, even after decades of management, points to the need for periodic soil testing and careful selection of inputs (fertilizers, pesticides, composts) to prevent long-term contamination [46,48]. Fourth, the dual-speed reassembly framework implies that short-term crop rotations may not adequately restore fungal communities if bacterial-driven nutrient fluctuations are too rapid. Allowing fallow periods or incorporating slower-growing cover crops could help balance the two microbial strategies [9,54]. Collectively, these implications advocate for an integrated soil management approach that combines biological monitoring (enzyme activities, network indicators), physical conservation (reduced tillage, structural protection), and chemical stewardship (input quality control) to enhance both short-term productivity and long-term soil health in botanical gardens and analogous intensive ornamental systems [76].

## 5. Conclusions

Intensive ornamental planting fundamentally reshapes soil microbial communities by altering both environmental conditions and interaction architectures. While bacterial diversity remains relatively stable, bacterial community composition and network structure respond sensitively to planting-driven disturbances, exhibiting increasing complexity and modular organization. In contrast, fungal communities are less responsive to short-term planting cycles but maintain structurally robust and compartmentalized interaction networks. These contrasting responses reveal a dual-speed reassembly mechanism in which bacterial networks capture short-term environmental fluctuations, whereas fungal networks encode longer-term habitat stability. Importantly, microbial network reassembly occurs primarily through interaction rewiring among persistent dominant taxa rather than taxonomic replacement. This decoupling of rapid functional responsiveness and structural stability suggests that intensive ornamental planting enhances microbial dynamism without necessarily compromising ecological resilience. Collectively, our findings underscore that soil nutrient pools and enzyme activities serve as critical determinants of soil health in horticultural systems in botanical gardens, mediating the balance between microbial responsiveness and structural stability under intensive management. These insights provide a foundation for integrating network-level perspectives into the sustainable design and management of urban green spaces.

## Figures and Tables

**Table 1:** Soil physicochemical properties and enzyme activities across site types and successive planting stages.

Parameters	L1	O1	L2	O2	L3	O3
pH	7.9 ± 0.1 c	7.8 ± 0.1 ab	7.9 ± 0 c	7.7 ± 0 a	7.7 ± 0.1 ab	7.8 ± 0 b
SOC (mg/kg)	255.2 ± 2.0 d	457.0 ± 6.4 a	229.5 ± 1.5 b	448.2 ± 5.5 c	227.4 ± 2.9 b	456.7 ± 7.3 a
TN (mg/kg)	22.1 ± 0.2 d	36.1 ± 0.3 a	21.4 ± 0.3 b	36.3 ± 0 ac	21.8 ± 0.1 bd	36.6 ± 0.7 c
AN (mg/kg)	169.3 ± 4.9 f	255.2 ± 5.4 a	151.2 ± 4.4 b	278.1 ± 8.0 c	128.3 ± 4.8 d	229.6 ± 6.3 e
TP (mg/kg)	8.5 ± 0.3 f	15.0 ± 0.3 a	8.2 ± 0 b	14.1 ± 0.1 c	7.6 ± 0 d	12.9 ± 0.2 e
AP (mg/kg)	39.8 ± 0.3 e	94.1 ± 1.4 a	34.2 ± 0.8 b	95.0 ± 1.8 a	29.5 ± 0.8 c	108.1 ± 0.7 d
TK (mg/kg)	224.5 ± 1.2 e	230.4 ± 3.1 a	229.7 ± 1.2 a	217.2 ± 1.1 b	226.7 ± 0.3 c	208.4 ± 1.1 d
AK (mg/kg)	160.5 ± 0.2 e	185.9 ± 2.2 a	173.5 ± 0.8 b	192.5 ± 1.6 c	175.4 ± 4.5 b	210.8 ± 6.7 d
Cd (mg/kg)	0.2 ± 0 b	0.2 ± 0 a	0.2 ± 0 b	0.3 ± 0 c	0.2 ± 0 d	0.2 ± 0 ad
Cu (mg/kg)	19.4 ± 0.4 d	25.7 ± 1.9 ab	23.2 ± 1.3 a	27.0 ± 0.7 bc	29.9 ± 5.5 c	24.3 ± 0.9 ab
Co (mg/kg)	9.2 ± 0.1 c	10.8 ± 1.4 a	10.9 ± 0.7 a	11.4 ± 0.6 ab	12.1 ± 0.1 b	10.9 ± 0.3 a
Cr (mg/kg)	52.9 ± 0.9 d	71.8 ± 7.7 a	62.3 ± 4.6 b	80.7 ± 2.8 c	69.0 ± 0.2 a	77.7 ± 3.3 c
Mn (mg/kg)	0.5 ± 0 c	0.6 ± 0 a	0.5 ± 0 b	0.5 ± 0 a	0.5 ± 0 a	0.5 ± 0 a
Ni (mg/kg)	23.8 ± 1.2 b	29.1 ± 3.2 a	29.9 ± 5.4 a	31.2 ± 1.0 a	30.6 ± 1.5 a	28.8 ± 0.6 a
Pb (mg/kg)	16.8 ± 0.2 b	18.2 ± 1.7 ab	18.9 ± 1.9 a	19.2 ± 0.8 a	21.8 ± 0.1 c	18.0 ± 0.1 ab
Zn (mg/kg)	77.4 ± 1.6 c	120.5 ± 20.4 a	98.7 ± 2.1 b	119.2 ± 3.2 a	100.2 ± 6.1 b	105.9 ± 5.5 b
V (mg/kg)	71.8 ± 1.3 b	77.3 ± 9.4 ab	82.6 ± 4.6 ac	86.4 ± 2.9 cd	90.1 ± 1.0 d	80.2 ± 1.3 a
As (mg/kg)	5.2 ± 0.2 ac	4.8 ± 1 a	5.8 ± 0.1 b	5.6 ± 0.6 bc	6.1 ± 0 b	4.8 ± 0.1 a
Mo (mg/kg)	0.7 ± 0 d	1.1 ± 0.1 a	0.9 ± 0.1 b	1.1 ± 0.2 a	1.1 ± 0 ac	1.2 ± 0 c
Sb (mg/kg)	0.5 ± 0 a	0.5 ± 0 a	0.7 ± 0.2 b	0.8 ± 0 b	0.9 ± 0 c	0.7 ± 0 b
Hg (mg/kg)	0.7 ± 0.1 bc	0.5 ± 0 a	0.6 ± 0.1 b	0.5 ± 0.1 a	0.7 ± 0 c	0.5 ± 0 a
BG (mg/g/24 h)	77.2 ± 0.6 a	78.0 ± 3.8 a	78.1 ± 2.1 a	76.2 ± 1.1 a	82.8 ± 2.4 b	66.9 ± 4.8 c
ACP (mg/g/24 h)	1.6 ± 0 d	1.8 ± 0.1 a	1.4 ± 0 b	1.7 ± 0 c	1.4 ± 0 b	1.9 ± 0 a
SUC (mg/g/24 h)	34.7 ± 2.6 d	40.4 ± 0.2 a	37.2 ± 1.7 b	41.7 ± 1.1 ac	38.1 ± 0.5 b	43.5 ± 1.2 c
URE (mg/g/24 h)	2.4 ± 0 c	2.7 ± 0 a	2.3 ± 0 bc	2.6 ± 0.1 a	2.3 ± 0.1 b	2.3 ± 0 bc
CAT (mg/g/24 h)	1292.5 ± 5.2 d	1016.2 ± 26.4 a	1412.1 ± 45.6 b	1058.7 ± 27.8 ac	1484.5 ± 87.8 b	1091.8 ± 88.3 c

Note: SOC, soil organic carbon; TN, total nitrogen; AN, alkaline hydrolyzable nitrogen; TP, total phosphorus; AP, available phosphorus; TK, total potassium; AK, available potassium; BG, β-1,4-glucosidase; ACP, acid phosphatase; SUC, sucrase; URE, urease; CAT, catalase. Values are presented as mean ± standard deviation. Different lowercase letters indicate significant differences among treatments (*p* < 0.05). O, ornamental planting area; L, lawn-tree area. Planting stages: tulip (L1/O1), summer annual ornamentals (L2/O2), chrysanthemum (L3/O3).

## Data Availability

The data supporting this study are openly available in the NCBI BioProject database at https://dataview.ncbi.nlm.nih.gov/object/PRJNA1433055 (accessed on 3 March 2026), under accession number PRJNA1433055.

## Supplementary Materials

The following supporting information can be downloaded at: https://www.mdpi.com/article/10.3390/microorganisms14040865/s1. Figure S1: Correlation heatmaps between environmental variables and microbial community composition at the phylum level for bacteria (a) and fungi (b). * *p* < 0.05; ** *p* < 0.01; Table S1: Relative abundances of dominant bacterial phyla across sites and planting stages; Table S2: Relative abundances of dominant fungal phyla across sites and planting stages; Table S3: PERMANOVA results showing the effects of site type and planting stage on bacterial community composition based on Bray–Curtis dissimilarity; Table S4: PERMANOVA results showing the interactive effects of site type and planting stage on bacterial community composition; Table S5: Pairwise PERMANOVA comparisons of bacterial community composition among planting stages; Table S6: PERMDISP tests for homogeneity of multivariate dispersions in bacterial communities between sites; Table S7: PERMDISP tests for homogeneity of multivariate dispersions in bacterial communities among planting stages; Table S8: PERMANOVA results showing the effects of site type and planting stage on fungal community composition based on Bray–Curtis dissimilarity; Table S9: PERMANOVA results showing the interactive effects of site type and planting stage on fungal community composition; Table S10: PERMDISP tests for homogeneity of multivariate dispersions in fungal communities between sites; Table S11: PERMDISP tests for homogeneity of multivariate dispersions in fungal communities among planting stages; Table S12: *p*-value of correlation between bacterial community composition and environmental factors; Table S13: *p*-value of correlation between fungal community composition and environmental factors; Table S14: Topological properties of bacterial co-occurrence networks across planting stages and sites; Table S15: Topological properties of fungal co-occurrence networks across planting stages and sites; Table S16: Taxonomic composition of nodes in bacterial co-occurrence networks; Table S17: Taxonomic composition of nodes in fungal co-occurrence networks.
