# Supplementary material for: Dual-Speed Reassembly of Soil Microbial Networks Under Intensive Ornamental Planting: Divergent Stability Strategies of Bacteria and Fungi in Botanical Garden Cinnamon Soils
Source: Microorganisms. 2026 Apr 11;14(4):865. doi: 10.3390/microorganisms14040865 (PMC13119146; doi:10.3390/microorganisms14040865)
Supplement: Supplementary file 1 [file microorganisms-14-00865-s001.zip › microorganisms-4244365-supplementary.pdf]

Dual-Speed Reassembly of Soil Microbial Networks Under Intensive Ornamental Planting: Divergent Stability Strategies of Bacteria and Fungi in Botanical Garden Cinnamon Soils

Tai Gao [1,2,3], Dakang Zhou [1,2,3], Baibing Wang [1,2,3], Ruifeng Wang [1,2,3], Gan Xiao [1,2,3], Han Quan [1,2,3] and Yu Wei [1,2,3,*]

[1] Beijing Botanical Garden Management Office, Bejing 100093, China; gaotai36@outlook.com (T.G.); zdk0812@163.com (D.Z.); wbb_bill@126.com (B.W.); feng952525@163.com (R.W.); xiaogan@chnbg.cn (G.X.); wangyindz@sina.com (H.Q.)

[2] Beijing Floriculture Engineering Technology Research Center, Bejing 100093, China;

[3] Key Laboratory of National Forestry and Grassland Administration on Plant Ex situ Conservation, Bejing 100093, China.

* Correspondence: weiyu@chnbg.cn (Y.W.); Tel.: +86-010-62591566

Table S1. Relative abundances of dominant bacterial phyla across sites and planting stages.

Phylum	L1	O1	L2	O2	L3	O3
Others	0.10 ± 0.01a	0.09 ± 0.02a	0.11 ± 0.02ab	0.09 ± 0.02a	0.12 ± 0.02b	0.10 ± 0.02a
Pseudomonadota	0.42 ± 0.03abc	0.45 ± 0.05ab	0.38 ± 0.04c	0.48 ± 0.03a	0.40 ± 0.07bc	0.45 ± 0.04ab
Actinomycetota	0.26 ± 0.04ab	0.26 ± 0.05ab	0.27 ± 0.06a	0.22 ± 0.03ab	0.20 ± 0.03b	0.20 ± 0.05b
Acidobacteriota	0.11 ± 0.02b	0.07 ± 0.01a	0.11 ± 0.04b	0.06 ± 0.02a	0.12 ± 0.04b	0.09 ± 0.02ab
Bacteroidota	0.04 ± 0.01ab	0.04 ± 0.01ab	0.04 ± 0.01ab	0.05 ± 0.01a	0.03 ± 0.01b	0.04 ± 0.02ab
Gemmatimonadota	0.02 ± 0c	0.03 ± 0ab	0.02 ± 0c	0.03 ± 0.01ab	0.03 ± 0.01ac	0.04 ± 0.01b
Bacillota	0.02 ± 0.01ab	0.03 ± 0a	0.02 ± 0.01b	0.03 ± 0.01a	0.01 ± 0.01b	0.02 ± 0.01b
Chloroflexota	0.02 ± 0ac	0.02 ± 0.01ab	0.01 ± 0c	0.01 ± 0c	0.02 ± 0.01ac	0.03 ± 0.01b
Verrucomicrobiota	0.01 ± 0a	0.01 ± 0a	0.01 ± 0.01ab	0.01 ± 0a	0.03 ± 0.01c	0.02 ± 0.01bc
Planctomycetota	0.01 ± 0ab	0.01 ± 0ab	0.01 ± 0a	0.01 ± 0b	0.02 ± 0.01c	0.01 ± 0.01ab
Myxococcota	0.01 ± 0a	0.01 ± 0a	0.01 ± 0ab	0.01 ± 0a	0.02 ± 0.01c	0.01 ± 0bc

* Values are presented as mean ± standard deviation. Different lowercase letters indicate significant differences among treatments ($p < 0.05$). O, ornamental planting area; L, lawn–tree area. Planting stages: tulip (L1/O1), summer annual ornamentals (L2/O2), chrysanthemum (L3/O3).

Table S2. Relative abundances of dominant fungal phyla across sites and planting stages.

Phylum	L1	O1	L2	O2	L3	O3
Others	0.15 ± 0.13a	0.08 ± 0.05a	0.13 ± 0.07a	0.12 ± 0.11a	0.15 ± 0.08a	0.08 ± 0.07a
Ascomycota	0.74 ± 0.12b	0.87 ± 0.06a	0.75 ± 0.06bc	0.80 ± 0.09abc	0.73 ± 0.08b	0.86 ± 0.07ac
Basidiomycota	0.06 ± 0.02bc	0.03 ± 0.01a	0.08 ± 0.01b	0.05 ± 0.04bc	0.07 ± 0.01b	0.03 ± 0.01ac
Mortierellomycota	0.04 ± 0.01c	0.02 ± 0.01ab	0.03 ± 0.01a	0.01 ± 0b	0.03 ± 0.01a	0.01 ± 0b
Rozellomycota	0.01 ± 0abc	0 ± 0a	0.01 ± 0.01bc	0 ± 0ab	0.01 ± 0c	0 ± 0ab
Mucoromycota	0 ± 0a	0 ± 0ab	0 ± 0a	0.01 ± 0c	0 ± 0a	0.01 ± 0b
Zoopagomycota	0 ± 0a	0 ± 0a	0 ± 0.01a	0 ± 0a	0.01 ± 0.01b	0 ± 0a
Blastocladiomycota	0 ± 0c	0 ± 0a	0 ± 0b	0 ± 0a	0 ± 0b	0 ± 0a
Chytridiomycota	0 ± 0bc	0 ± 0a	0 ± 0ab	0 ± 0a	0 ± 0c	0 ± 0a
Aphelidiomycota	0 ± 0ab	0 ± 0ab	0 ± 0a	0 ± 0ab	0 ± 0a	0 ± 0b

* Values are presented as mean ± standard deviation. Different lowercase letters indicate significant differences among treatments ($p < 0.05$). O, ornamental planting area; L, lawn-tree area. Planting stages: tulip (L1/O1), summer annual ornamentals (L2/O2), chrysanthemum (L3/O3).

Table S3. PERMANOVA results showing the effects of site type and planting stage on bacterial community composition based on Bray-Curtis dissimilarity.

Factor	Df	SumOfSqs	R^2	F	Pr(>F)	sig
Site	1	0.170	0.168	14.471	0.001	***
Stage	2	0.183	0.180	7.758	0.001	***
Residual	56	0.659	0.651			
Total	59	1.011	1.000			

Table S4. PERMANOVA results showing the interactive effects of site type and planting stage on bacterial community composition.

Factor	Df	SumOfSqs	R^2	F	Pr(>F)
Site:Stage	2	0.024	0.024	1.032	0.392
Residual	54	0.635	0.627		
Total	59	1.012	1		

* PERMANOVA was performed using Bray-Curtis dissimilarity matrices derived from phylum-level relative abundance data. Statistical significance was assessed with 999 permutations.

Table S5. Pairwise PERMANOVA comparisons of bacterial community composition among planting stages.

Pairs	Df	SumsOfSqs	F.Model	R^2	p.value	p.adjusted	sig
Tulip vs Summer	1	0.012	0.851	0.022	0.461	0.461	
Tulip vs Chrysanthemum	1	0.131	11.148	0.227	0.001	0.002	*
Summer vs Chrysanthemum	1	0.130	7.439	0.164	0.001	0.002	*

Table S6. PERMDISP tests for homogeneity of multivariate dispersions in bacterial communities between sites.

Factor	Df	Sum Sq	Mean Sq	F	N.Perm	Pr(>F)
Groups	1	0.004	0.004	1.501	999	0.192
Residuals	58	0.171	0.003			

Table S7. PERMDISP tests for homogeneity of multivariate dispersions in bacterial communities among planting stages.

Factor	Df	Sum Sq	Mean Sq	F	N.Perm	Pr(>F)	sig
Groups	2	0.021	0.011	5.619	999	0.004	**
Residuals	57	0.108	0.002				

Table S8. PERMANOVA results showing the effects of site type and planting stage on fungal community composition based on Bray-Curtis dissimilarity.

Factor	Df	SumOfSqs	R^2	F	Pr(>F)	sig
Site	1	0.179	0.225	16.775	0.001	***
Stage	2	0.018	0.023	0.843	0.461	
Residual	56	0.599	0.752			
Total	59	0.796	1			

Table S9. PERMANOVA results showing the interactive effects of site type and planting stage on fungal community composition.

Factor	Df	SumOfSqs	R^2	F	Pr(>F)
Site:Stage	2	0.046615	0.05855	2.279433	0.058
Residual	54	0.552154	0.693531		
Total	59	0.796149	1		

* PERMANOVA was performed using Bray-Curtis dissimilarity matrices derived from phylum-level relative abundance data. Statistical significance was assessed with 999 permutations.

Table S10. PERMDISP tests for homogeneity of multivariate dispersions in fungal communities between sites.

Factor	Df	Sum Sq	Mean Sq	F	N.Perm	Pr(>F)
Groups	1	0.0004	0.0004	0.083	999	0.77
Residuals	58	0.282	0.005			

Table S11. PERMDISP tests for homogeneity of multivariate dispersions in fungal communities among planting stages.

Factor	Df	Sum Sq	Mean Sq	F	N.Perm	Pr(>F)
Groups	2	0.0005	0.0002	0.051	999	0.953
Residuals	57	0.280	0.005			

Table S12. P value of correlation between bacterial community composition and environmental factors.

Parameters	r^2	p
SUC	0.5140	0.0005
Hg	0.4833	0.0005
Mn	0.3347	0.0005
Zn	0.3191	0.0005
pH	0.2652	0.0010
BG	0.2555	0.0010
As	0.1862	0.0030
Cu	0.1019	0.0425

* SUC, sucrase; BG, β-1,4-glucosidase; O, ornamental planting area; L, lawn-tree area. Planting stages: tulip (L1/O1), summer annual ornamentals (L2/O2), chrysanthemum (L3/O3).

Table S13. P value of correlation between fungal community composition and environmental factors.

Parameters	r^2	p
SUC	0.6760	0.0005
Zn	0.5144	0.0005
Hg	0.4910	0.0005
Mn	0.4611	0.0005
As	0.4262	0.0005
Ni	0.4231	0.0005
Cu	0.3419	0.0005
pH	0.3244	0.0005
BG	0.2546	0.0005

* SUC, sucrase; BG, β-1,4-glucosidase; O, ornamental planting area; L, lawn-tree area. Planting stages: tulip (L1/O1), summer annual ornamentals (L2/O2), chrysanthemum (L3/O3).

Table S14. Topological properties of bacterial co-occurrence networks across planting stages and sites.

Network Topology	L1	O1	L2	O2	L3	O3
Number of nodes	55	32	41	108	88	186
Number of edges	32	16	22	102	55	142
Positive edges	32	14	20	99	53	136
Negative edges	0	2	2	3	2	6
Graph density	0.022	0.032	0.027	0.018	0.014	0.008
Average degree	1.16	1	1.07	1.89	1.25	1.53
Modularity	0.943	0.938	0.942	0.859	0.963	0.949

Table S15. Topological properties of fungal co-occurrence networks across planting stages and sites.

Network Topology	L1	O1	L2	O2	L3	O3
Number of nodes	107	58	76	48	97	50
Number of edges	119	47	76	45	77	34
Positive edges	118	47	76	44	76	34
Negative edges	1	0	0	1	1	0
Graph density	0.021	0.028	0.027	0.040	0.017	0.028
Average degree	2.22	1.62	2	1.88	1.59	1.36
Modularity	0.879	0.909	0.93	0.852	0.949	0.939

Table S16. Taxonomic composition of nodes in bacterial co-occurrence networks.

L1	O1	L2	O2	L3	O3
Acidobacteriota (34.55%)	Pseudomonadota (28.12%)	Pseudomonadota (31.71%)	Pseudomonadota (25.93%)	Acidobacteriota (30.68%)	Acidobacteriota (24.73%)
Actinomycetota (21.82%)	Actinomycetota (21.88%)	Acidobacteriota (24.39%)	Acidobacteriota (25.00%)	Pseudomonadota (23.86%)	Pseudomonadota (24.19%)
Pseudomonadota (21.82%)	Acidobacteriota (18.75%)	Actinomycetota (21.95%)	Actinomycetota (15.74%)	Planctomycetota (10.23%)	Actinomycetota (11.29%)
Planctomycetota (9.09%)	Chloroflexota (9.38%)	Thermodesulfobacteriota (2.44%)	Gemmatimonadota (6.48%)	Actinomycetota (9.09%)	Planctomycetota (8.06%)
Myxococcota (7.27%)	Planctomycetota (6.25%)	Planctomycetota (2.44%)	Chloroflexota (4.63%)	Myxococcota (4.55%)	Gemmatimonadota (5.91%)
Bacteroidota (3.64%)	Gemmatimonadota (6.25%)	Gemmatimonadota (2.44%)	Methylomirabilota (3.7%)	Chloroflexota (4.55%)	Chloroflexota (4.84%)
Other (1.82%)	Nitrospirota (3.12%)	Verrucomicrobiota (2.44%)	Thermoproteota (3.7%)	Methylomirabilota (3.41%)	Myxococcota (3.76%)
	Other (6.25%)	Other (12.20%)	Planctomycetota (2.78%)	Verrucomicrobiota (2.27%)	Latescibacterota (2.69%)
			Bacteroidota (2.78%)	Entotheonellaeota (1.14%)	Thermoproteota (2.69%)
			Latescibacterota (1.85%)	Thermoproteota (1.14%)	Verrucomicrobiota (2.69%)
			Bacillota (1.85%)	Nitrospirota (1.14%)	Methylomirabilota (2.15%)
			Thermodesulfobacteriota (0.93%)	Latescibacterota (1.14%)	Bacteroidota (1.61%)
			Myxococcota (0.93%)	Gemmatimonadota (1.14%)	Thermodesulfobacteriota (1.08%)
			Other (3.70%)	Other (5.68%)	Nitrospirota (0.54%)
					Entotheonellaeota (0.54%)
					Bacillota (0.54%)
					Armatimonadota (0.54%)
					Other (2.16%)

* O, ornamental planting area; L, lawn-tree area. Planting stages: tulip (L1/O1), summer annual ornamentals (L2/O2), chrysanthemum (L3/O3).

Table S17. Taxonomic composition of nodes in fungal co-occurrence networks.

L1	O1	L2	O2	L3	O3
Ascomycota (56.07%)	Ascomycota (68.97%)	Ascomycota (34.21%)	Ascomycota (58.33%)	Ascomycota (46.39%)	Ascomycota (52.00%)
Zoopagomycota (8.41%)	Basidiomycota (8.62%)	Basidiomycota (10.53%)	Basidiomycota (8.33%)	Zoopagomycota (8.25%)	Basidiomycota (10.00%)
Basidiomycota (5.61%)	Mortierellomycota (1.72%)	Zoopagomycota (7.89%)	Blastocladiomycota (2.08%)	Basidiomycota (6.19%)	Aphelidiomycota (2.00%)
Mortierellomycota (3.74%)	Mucoromycota (1.72%)	Mortierellomycota (1.32%)	Mucoromycota (2.08%)	Rozellomycota (2.06%)	Other (36.00%)
Chytridiomycota (1.87%)	Other (18.96%)	Blastocladiomycota (1.32%)	Neocallimastigomycota (2.08%)	Mortierellomycota (1.03%)	
Other (24.30%)		Mucoromycota (1.32%)	Other (27.08%)	Glomeromycota (1.03%)	
		Rozellomycota (1.32%)		Blastocladiomycota (1.03%)	
		Other (42.10%)		Mucoromycota (1.03%)	
				Olpidiomycota (1.03%)	
				Other (31.96%)	

* O, ornamental planting area; L, lawn-tree area. Planting stages: tulip (L1/O1), summer annual ornamentals (L2/O2), chrysanthemum (L3/O3).

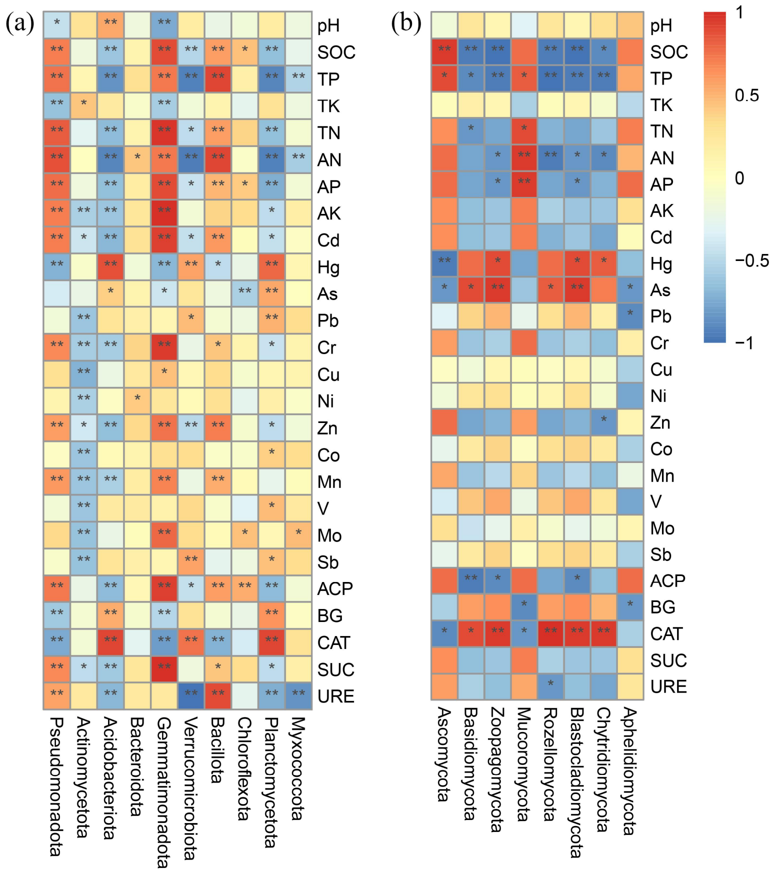

Figure S1. Correlation heatmaps between environmental variables and microbial community composition at the phylum level for bacteria (a) and fungi (b). * $p < 0.05$; ** $p < 0.01$.
